# Exploring cranial macromorphoscopic variation and classification accuracy in a South African sample

**DOI:** 10.1007/s00414-024-03230-2

**Published:** 2024-04-16

**Authors:** Leandi Liebenberg, Ericka N. L’Abbé, Kyra E. Stull

**Affiliations:** 1 Department of Anatomy, University of Pretoria, Private Bag x323, Arcadia, 0007 South Africa; 2 Forensic Anthropology Research Centre, University of Pretoria, Arcadia, South Africa; 3 Department of Anthropology, University of Nevada, Reno, USA

**Keywords:** Forensic anthropology, Population affinity, Ancestry, Random forest, Variable importance

## Abstract

To date South African forensic anthropologists are only able to successfully apply a metric approach to estimate population affinity when constructing a biological profile from skeletal remains. While a non-metric, or macromorphoscopic approach exists, limited research has been conducted to explore its use in a South African population. This study aimed to explore 17 cranial macromorphoscopic traits to develop improved methodology for the estimation of population affinity among black, white and coloured South Africans and for the method to be compliant with standards of best practice. The trait frequency distributions revealed substantial group variation and overlap, and not a single trait can be considered characteristic of any one population group. Kruskal-Wallis and Dunn’s tests demonstrated significant population differences for 13 of the 17 traits. Random forest modelling was used to develop classification models to assess the reliability and accuracy of the traits in identifying population affinity. Overall, the model including all traits obtained a classification accuracy of 79% when assessing population affinity, which is comparable to current craniometric methods. The variable importance indicates that all the traits contributed some information to the model, with the inferior nasal margin, nasal bone contour, and nasal aperture shape ranked the most useful for classification. Thus, this study validates the use of macromorphoscopic traits in a South African sample, and the population-specific data from this study can potentially be incorporated into forensic casework and skeletal analyses in South Africa to improve population affinity estimates.

## Introduction

The parameters of the biological profile consist of estimations of age-at-death, stature, sex and population affinity, and require knowledge of skeletal variation within and between populations to be accurately established. Populations are groups with diverse histories influenced by numerous factors, all of which contribute to the patterned distribution of human variation [1, 2]. The quantification of skeletal variation among populations forms the basis of population affinity, where the estimation of population affinity is considered possible as skeletal variation has been correlated to socially constructed populations around the world [1]. However, the relationship between skeletal morphology and social race is complicated and is important to acknowledge [3]. This inherent complexity should be considered in all aspects of research, including terminology and method design, and in drawing conclusions when attempting to quantify population variation from the skeleton [4]. Forensic anthropologists are attempting to be more cognizant of this fact and aim to enact transformation in how population variation is described and explored in the discipline.

The cranium is often considered the most accurate skeletal element for the evaluation of population affinity, with craniometry elected as the preferred approach. Numerous studies have assessed craniometric variation among South Africans [e.g., 5–4]. The use of standard craniometric variables have been found to produce satisfactory results when estimating population affinity with correct classifications up to 73% [7]. However, standard linear measurements mainly quantify size and are frequently unable to effectively capture the shape variation observable in the craniofacial complex. The use of alternative metric methods, such as geometric morphometrics, has gained greater popularity amid anthropological research [10]. Geometric morphometrics entails recording landmark coordinates of complex objects in a three-dimensional space which then produces statistical and graphical outputs primarily using shape information. Shape differences among specimens can be observed as displacement of individual landmarks within the total configuration of the object being assessed [11]. Researchers have noted coordinate-based analyses achieve greater classification accuracies than standard linear metrics, with approximately 89% correct classifications among three modern South African groups [8]. Thus, shape variation is of great importance when exploring craniofacial morphology and its use in assessing population affinity. The application of non-metric visual assessment is an alternative to quantify cranial size as well as shape in instances where geometric morphometric techniques are not a feasible option, as the method does not require any equipment and is not time consuming. However, the use of non-metrics is associated with numerous methodological issues and is known for perpetuating racial typological thinking in the assessment and understanding of human variation [12, 13]. As such, emerging research around the world has attempted to challenge and to improve the non-metric approach, now referred to as the macromorphoscopic (MMS) method, inclusive of adding definitions and comparative drawings, employing robust statistical tests, and gauging the accuracy of the method in different populations [12–15]. Greater emphasis has also been placed on exploring observer agreement and trait score variation when employing the traits [16, 17].

To date the MMS method has yet to undergo the same level of application and rigorous scientific testing in South Africa. While the frequency of some of the traits have been assessed, its application in classification models for the purpose of forensic analyses has been very limited [18, 19]. With a lack of population-specific standards, South African practitioners may rely on North American standards, which is not recommended as differences have been shown to exist between North Americans and South Africans [18, 20–22]. This requires for additional work to be done to ensure the method meets international standards for best scientific practice [23]. The purpose of this study was to explore the MMS cranial variation among black, white and coloured South Africans to improve the methodology employed to estimate population affinity.

## Materials and methods

The sample consisted of 660 crania of black, white and coloured South Africans (Table 1). The South African population is diverse and consists of four major groups: South African blacks (81.0%), whites (7.7%) and coloureds (8.8%) make up the majority of the population; the remaining 2.6% of the population consists of individuals classified as Asian and Indian [24] (Statistics South Africa, 2022). Each group has a unique history within the country leading to the vast heterogeneity observed within and among the groups. Black South Africans descend from Bantu-speaking groups that migrated throughout sub-Saharan Africa from western-central Africa approximately 3000 to 5000 years ago [25]. Further divisions among the southern Bantu-speakers based on factors associated with kinship, religion and language resulted in the numerous subgroups residing in southern Africa today [26]. Colonization of the Cape during the 17th century introduced European settlers to South Africa, shaping the heritage of white South Africans. The settlers were mainly of Dutch origin, with additional contributions from French Huguenots and Germans that arrived in the 18th century. Late in the 18th century South Africa was also colonized by the English [27]. Coloured South African refers to a self-identified group unique to South Africa. The group is a result of the complex history of South Africa with genetic contributions from Khoe-San (considered indigenous South Africans), Bantu-speakers, Europeans, as well as Indians and other Asian groups that were brought to South Africa as slaves to maintain the Cape colony. The complex population structure and history of the coloured South Africans manifests as a genetically and skeletally heterogeneous group with substantial variation [8]. While the varying origins of each group resulted in a uniquely heterogeneous population with distinct structures, the group differences employed to attempt population affinity estimations persisted as a result of socio-political boundaries. Sociocultural identity in South Africa is based on the categorizations assigned to individuals during the *Apartheid* era, which contributed to widespread endogamy among groups [28].

The crania were sampled from the Pretoria Bone Collection (University of Pretoria) and the Kirsten Collection (Stellenbosch University) in South Africa. The remains accessioned into the collections are of documented sex, age at death, and peer-reported population affinity [29, 30]. Ethical approval (770/2018) to conduct the study was obtained from the Faculty of Health Sciences Research Ethics Committee at the University of Pretoria.

**Table 1:** Sample distribution

Population	Males	Females	Total
Black SA	110	110	220
White SA	110	110	220
Coloured SA	110	110	220
Total	330	330	660

A total of 17 MMS traits were visually assessed and scored following the methodology described by Hefner [12] and Plemons and Hefner [13] as used in the Macromorphoscopic Traits collection module (MMS version 1.6.1) (Table 2). The MMS module was used to capture the scores for each individual. Where traits are bilaterally expressed, only the left side was recorded. If the left side was not available, the right side was used.

**Table 2:** Macromorphoscopic traits and abbreviations

Anterior nasal spine	ANS	Nasofrontal suture	NFS
Inferior nasal aperture	INA	Orbital shape	OS
Interorbital breadth	IOB	Post-bregmatic depression	PBD
Malar tubercle	MT	Posterior zygomatic tubercle	PZT
Nasal aperture shape	NAS	Supranasal suture	SPS
Nasal aperture width	NAW	Transverse palatine suture	TPS
Nasal bone contour	NBC	Palate shape	PS
Nasal bone shape	NBS	Zygomaticomaxillary suture	ZS
Nasal overgrowth	NO		

All statistical analyses were completed using the software R version 4.1.0 [31], and included assessments of observer agreement, exploratory analyses, and the creation of classification models. Ten crania were randomly selected to test observer agreement. Two observers scored the crania; both observers are experienced with skeletal analyses, but only one observer has extensive experience with the traits. The observers discussed the trait definitions and methodology prior to collecting the scores for analysis. The repeatability of the traits was assessed with Cohen’s kappa using the *irr* package in R; different weights were given to the scores depending on the data structure of the trait. Standard, unweighted kappa was used for the ordinal scores where the different trait states are unranked. For the ranked scores (i.e., ANS, INA, MT, NAW, and PZT), a quadratic weighted kappa was employed. Calculated kappa values can range from − 1 to 1, where values closer to 1 indicate greater agreement. No universally accepted cut-off point for satisfactory observer agreement currently exists. However, to be consistent with nomenclature when describing the strength of agreement associated with kappa statistics, the parameters proposed by Landis and Koch [32] was used.

The MMS scores were used to create frequency distributions to assess the occurrence of each trait per group. Kruskal-Wallis tests were used to identify if any traits demonstrated significant differences among the populations. Kruskal-Wallis is a non-parametric test used to compare three or more groups which operates under the assumptions of independence of scores but is not bound by assumptions of normality or homogeneity of variance [33]. Additionally, a *post-hoc* Dunn’s multiple comparisons test (with a Holm’s adjustment) was used to further explore differences in the trait frequencies among the populations. The Holm’s adjustment counteracts the effects of multiple comparisons and prevents increased probability of Type I errors occurring [34]. More specifically, where Kruskal-Wallis indicates the presence of significant differences, the Dunn’s test indicates which groups in a multiple comparison differ from one another to better interpret group overlap.

Random forest models (RFM) were created to classify the crania according to population affinity, as well as population affinity and sex concurrently. RFM is a non-parametric machine learning method that was introduced as an improvement upon decision trees [35]. Decision trees are a type of classification model that uses sequential splitting values (such as MMS traits) to predict the probability of an unknown belonging to a certain class (i.e., population affinity) to separate a dataset into groups [36]. Within each data split, known as “nodes” in the tree, the variable that is most strongly associated with the response variable (a specific group) is selected for the next split until a stopping condition is met. In the case of the current study, the stopping condition is an overall population estimate based on the ensemble of multivariate trees. The overall population estimate is reached by combining the most likely response from all of the nodes, or in the case of RFM, all of the trees in the ensemble. This is achieved by means of voting in classification; simply put, the population group that receives the most “votes” from the trees is returned as the overall prediction [35]. A total of 2500 classification trees were used for each model with four variables at each split. Furthermore, RFM ranks the importance of each variable included in the classification ensemble, giving an indication of which variables are most discriminatory in the model and which variables do not contribute to the classification [14]. Two measures of variable importance were employed, namely the mean decrease in the Gini index, and the mean decrease in the permutation accuracy. The Gini index measures how much each predictor variable contributes to the overall reduction in node impurity achieved by splitting the data on each variable across all trees in the forest. The mean decrease is calculated for each variable by averaging the reduction in the Gini index across all nodes where that specific variable is used for splitting. The Gini criterion has been shown to favour variables that have many categories (or trait states) and can be influenced by highly correlated variables; thus, the Gini index should not be used as the only indicator of variable importance [37]. The mean decrease in the permutation accuracy was also assessed, where the relative importance of each predictor variables is calculated by measuring the decrease in model accuracy across all trees upon removal of the variable. With both measures of variable importance, the higher the value, the more a variable contributes to the classification (i.e. the more important a variables is to the model). Finally, out-of-bag observations can be used to gauge the external prediction accuracy of the tree (comparable to leave-one-out cross-validation commonly used with discriminant analysis). The original training data is randomly sampled with replacement for each tree, which generates a smaller subset of data for each tree; essentially this is the training data. The observations excluded from the training data, or the out-of-bag observations, are a random subset of data that is essentially an internal test sample. The tree will then be used to classify the test sample to obtain a more realistic classification accuracy [38]. In the case of missing data, the mode was calculated for each trait per each sex and population group separately. The mode was used as an imputation value specifically because it appears the most in a set of values which in this case, is a population and sex group, most individuals are likely to depict that value. Data imputation was only performed when variables had less than 10% of the observations missing. For variables where more than 10% of the observations would have to be replaced, the variable was omitted from the model. After the missing data were imputed, the sample was divided so that 75% was used as the training set to create the model, and the remaining 25% was the holdout set to test the accuracy of the model on an independent set of crania. The *randomForest* package was used to generate the RFM classifications [39].

## Results

The intra-observer agreement ranged from 0.41 (moderate) to 1.00 (perfect), with nasal overgrowth (NO) and transverse palatine suture (TPS) performing the worst and best, respectively (Table 3). Following the descriptions proposed by Landis and Koch [32] eight out of the seventeen traits demonstrated substantial agreement, while six were observed to be almost perfect. The inter-observer agreement was overall lower, ranging between 0.11 (slight) and 0.91 (almost perfect). The traits that performed poorly varied between the observers. Since all of the data was collected by the first author (LL), the repeatability was considered acceptable, and all traits were retained for further analyses.

**Table 3:** Kappa values for inter- and intra-observer agreement. Bold indicates substantial agreement or higher following Landis and Koch [32]

	Intra-observer agreement	Inter-observer agreement
ANS	**0.82**	**0.66**
INA	0.47	**0.86**
IOB	**0.83**	**0.91**
MT	**0.72**	0.59
NAS	**0.62**	0.24
NAW	**0.91**	**0.91**
NBC	**0.64**	0.13
NBS	0.43	0.44
NO	0.41	**0.78**
NFS	**0.83**	**0.67**
OS	**0.80**	0.57
PBD	**0.74**	0.29
PZT	**0.69**	**0.72**
SPS	**0.81**	0.11
TPS	**1.00**	0.47
PS	**0.71**	0.18
ZS	**0.74**	**1.00**
*Mean*	0.72	0.56
*Min*	0.41	0.11
*Max*	1.00	0.91

Table 4 presents the frequencies for the MMS traits. The sample size varies for each trait because of the presence of post-mortem damage, ante-mortem trauma, and tooth loss. A substantial amount of group overlap was observed for the traits, and not a single trait can be considered characteristic of a population. Kruskal-Wallis tests were used to identify potential population group differences (Table 5). Overall, 13 out of the 17 traits were noted to differ significantly among the population groups (*p* < 0.05). The nasal bone shape (NBS), supra-nasal suture (SPS), transverse palatine suture (TPS), and palate shape (PS) did not differ significantly between the groups. Since Kruskal-Wallis only indicates if there are any differences, a *post-hoc* Dunn’s test was then used to further explore the variation among the three populations (see Table 6 for a breakdown of the group overlap). Five traits demonstrate no significant overlap among any of the groups; this includes the inferior nasal margin (INA), malar tubercle (MT), nasal aperture shape (NAS), nasal bone contour (NBC), and zygomaticomaxillary suture (ZS). The remainder of the traits demonstrated overlap between at least two of the groups. Black and coloured South Africans were observed to overlap more frequently, with some traits also presenting with overlap between coloured South Africans and white South Africans. However, none of the traits indicate significant overlap between black South Africans and white South Africans, suggesting the two groups are most dissimilar from one another. While coloured South Africans often overlapped with black South Africans, the coloured group more frequently yielded intermediate scores rather than extreme scores. Seven of the traits also demonstrated significant differences between the sexes (Table 5).

**Table 4:** Trait frequencies for the three population groups. Refer to Table 2 for trait abbreviations

	Population group					
	Black		Coloured		White	
Trait scores	n	%	n	%	n	%
**ANS**	(*N* = 220)		(*N* = 212)		(*N* = 207)	
1	143	65.0	115	54.2	25	12.1
2	66	30.0	85	40.1	79	38.2
3	11	5.0	12	5.7	103	49.7
**INA**	(*N* = 220)		(*N* = 219)		(*N* = 220)	
1	53	24.1	7	3.2	0	0.0
2	79	35.9	36	16.4	3	1.4
3	74	33.6	118	56.5	38	17.3
4	9	4.1	47	21.5	107	48.6
5	5	2.3	11	5.0	72	32.7
**IOB**	(*N* = 220)		(*N* = 219)		(*N* = 220)	
1	23	10.5	33	15.1	134	60.9
2	99	45.0	99	45.2	77	35.0
3	98	44.5	87	39.7	9	4.1
**MT**	(*N* = 218)		(*N* = 214)		(*N* = 220)	
0	2	1.0	0	0.0	16	7.3
1	116	53.2	151	70.6	167	75.9
2	75	34.4	59	27.6	34	15.5
3	25	11.5	4	1.9	3	1.4
**NAS**	(*N* = 220)		(*N* = 218)		(*N* = 220)	
1	28	12.7	65	29.8	183	83.2
2	36	16.4	17	7.8	28	12.7
3	156	70.9	136	62.4	9	4.1
**NAW**	(*N* = 220)		(*N* = 219)		(*N* = 220)	
1	5	2.3	6	2.7	80	36.4
2	67	30.5	74	33.8	113	51.4
3	148	67.3	139	63.5	27	12.2
**NBC**	(*N* = 194)		(*N* = 187)		(*N* = 202)	
0	116	59.8	70	37.4	0	0.0
1	44	22.7	87	46.5	39	19.3
2	7	3.6	7	3.7	79	39.1
3	9	4.6	14	7.5	78	38.6
4	18	9.3	9	4.8	6	3.0
**NBS**	(*N* = 213)		(*N* = 204)		(*N* = 214)	
1	58	27.2	25	12.3	32	15.0
2	107	50.2	153	75.4	167	78.0
3	26	12.2	7	3.4	12	5.6
4	22	10.3	18	8.9	3	1.4
**NO**	(*N* = 208)		(*N* = 186)		(*N* = 205)	
0	202	97.1	186	100.0	168	82.0
1	6	2.9	0	0.0	37	18.0
**NFS**	(*N* = 202)		(*N* = 200)		(*N* = 214)	
1	73	36.1	96	48.0	123	57.5
2	71	35.1	58	29.0	38	17.8
3	17	8.4	16	8.0	23	10.7
4	41	20.3	30	15.0	30	14.0
**OS**	(*N* = 219)		(*N* = 218)		(*N* = 220)	
1	118	53.9	159	72.9	150	68.2
2	89	40.6	44	20.2	49	22.3
3	12	5.5	15	6.9	21	9.5
**PBD**	(*N* = 218)		(*N* = 214)		(*N* = 217)	
0	144	65.1	155	72.4	176	81.1
1	74	33.9	59	27.6	41	18.9
**PZT**	(*N* = 218)		(*N* = 217)		(*N* = 220)	
0	14	6.4	6	2.8	25	11.4
1	77	35.3	65	30.0	104	47.3
2	72	33.0	91	41.9	63	28.6
3	55	25.2	55	25.3	28	12.7
**SPS**	(*N* = 219)		(*N* = 220)		(*N* = 220)	
0	69	31.5	29	13.2	23	10.5
1	19	8.7	85	38.6	89	40.5
2	131	59.8	106	48.2	108	49.0
**TPS**	(*N* = 213)		(*N* = 211)		(*N* = 215)	
1	53	24.9	54	25.6	59	27.4
2	110	51.6	119	56.4	126	58.6
3	23	10.8	15	7.1	14	6.5
4	27	12.7	23	10.9	16	7.5
**PS**	(*N* = 168)		(*N* = 116)		(*N* = 53)	
1	50	29.8	31	26.7	25	47.2
2	29	17.3	18	15.5	9	17.0
3	54	32.1	55	47.4	11	20.8
4	35	20.8	12	10.3	8	15.1
**ZS**	(*N* = 210)		(*N* = 209)		(*N* = 215)	
0	153	72.9	84	40.2	75	34.9
1	45	21.4	123	58.8	112	52.1
2	12	5.7	2	1.0	28	13.0

**Table 5:** Kruskal-Wallis test comparing trait score frequencies among the populations and between the sexes. Bold indicates significant differences

Trait	Population	Sex
ANS	**< 0.05**	0.08
INA	**< 0.05**	**< 0.05**
IOB	**< 0.05**	**< 0.05**
MT	**< 0.05**	**< 0.05**
NAS	**< 0.05**	0.76
NAW	**< 0.05**	**< 0.05**
NBC	**< 0.05**	0.05
NBS	0.28	0.24
NO	**< 0.05**	0.33
NFS	**< 0.05**	0.33
OS	**< 0.05**	0.18
PBD	**< 0.05**	0.07
PZT	**< 0.05**	**< 0.05**
SPS	0.92	**< 0.05**
TPS	0.19	0.93
PS	0.06	**< 0.05**
ZS	**< 0.05**	0.99

**Table 6:** Break down of group overlap for trait scores based on the Kruskal-Wallis and Dunn’s tests

No groups overlap	All groups overlap	B and C overlap	B and W overlap	W and C overlap
INA	NBS	ANS	-	NFS
MT	SPS	IOB		OS
NAS	TPS	NAW		PBD
NBC	PS	NO		
ZS		PBD		
		PZT		

All of the traits were combined into a multivariate classification model and the positive predictive performance was assessed using RFM. Given the substantial amount of missing data, palate shape (PS) was omitted from further analyses. Overall, the MMS traits yielded an accuracy of 78.7% when assessing population affinity. Table 7 presents the training accuracies, with a breakdown of the predictive performance of each population group and group overlap. The greatest overlap (and subsequent misclassification) was observed between black and coloured South Africans. White South Africans demonstrated the least overlap, resulting in the highest group accuracy (89.7%). The testing model (which serves as an independent validation) yielded an overall accuracy of 81.8%.

**Table 7:** Confusion matrix showing patterns of overlap and misclassification among the groups for the training model employing the MMS traits

		Classifies into:			% Correct
		B	W	C	
Group:	B	127	5	33	77.0
	W	3	148	14	89.7
	C	32	18	115	69.7
Total:					78.7

**Fig. 1:**
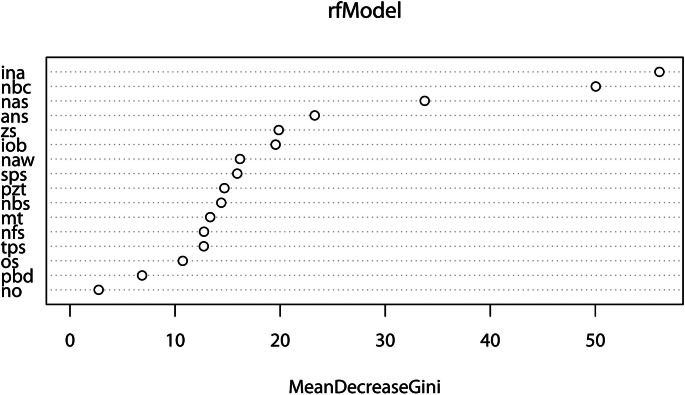
Variable importance (based on the mean decrease in the Gini index) for the multivariate model assessing population affinity employing all MMS traits

The variable importance was calculated to assess the amount of discriminatory power each trait contributes to the model and overall correct classification. Ultimately all traits contributed some information to the model. The mean decrease in the Gini index ranged from 2.7 to 56.0, with the mean decrease in the permutation accuracy ranging between 0.0 and 12.9% (Table 8). Figure 1 graphically demonstrates the contribution of each trait to the model based on the Gini index. The highest ranked traits for both measures of variable importance include the inferior nasal margin (INA), nasal bone contour (NBC), and nasal aperture shape (NAS) – i.e., variables in the nasal region. The lowest ranked traits include nasal overgrowth (NO) and post-bregmatic depression (PBD). Additional models were created where the number of traits were systematically reduced; more specifically, traits with poor repeatability as noted with Cohen’s kappa, any trait that did not yield significant differences with Kruskal-Wallis, and any trait with low variable importance were removed and the models were run again. A reduction in the number of traits in the model consistently yielded decreased classification accuracies, suggesting that all traits be retained in analyses for optimal results.

**Table 8:** RFM variable importance for MMS model assessing population affinity

Trait	Mean Gini decrease	Mean accuracy decrease (%)
INA	56.0	12.9
NBC	50.0	11.7
NAS	33.8	6.3
ANS	23.3	2.6
ZS	19.9	1.9
IOB	19.6	2.2
NAW	16.2	1.8
SPS	15.9	3.6
PZT	14.7	1.2
NBS	14.4	1.4
MT	13.3	1.2
NFS	12.8	1.2
TPS	12.7	1.6
OS	10.7	2.2
PBD	6.9	0.6
NO	2.7	0.0

Since a number of traits also indicated a significant relationship with sex, RFM was used to assess the accuracy with which both population affinity and sex can be classified concurrently. With classification among six groups (black males and females, white males and females, and coloured males and females), the training model yielded an accuracy of 57.7% (Table 9), while the testing model yielded and accuracy of 61.7%. Overall, the individuals were frequently classified into the correct population groups, but misclassified more frequently according to sex. Coloured females presented with the lowest group accuracy (47.0%), with increased instances of misclassification into the incorrect population group as well as the incorrect sex.

**Table 9:** Confusion matrix showing patterns of overlap and misclassification among the groups and sexes for the training model employing the MMS traits

		Classifies into:						% Correct
		BM	BF	WM	WF	CM	CF	
Group:	BM	45	15	2	0	13	8	54.2
	BF	19	45	1	2	4	12	54.2
	WM	1	0	58	20	3	1	69.9
	WF	0	0	22	50	1	10	60.2
	CM	10	2	5	1	49	19	59.1
	CF	9	12	0	7	16	39	47.0
Total:								57.7

Similar patterns of overlap were observed when sex-specific analyses were conducted (i.e., comparing population groups but with the sexes separated) (Table 10). The sex-specific analysis comparing males yielded a greater accuracy (83.5%) than the model with the sexes pooled (78.7%), while the female sex-specific analysis yielded a slightly lower accuracy (76.7%). Although the coloured females still demonstrate the lowest classification accuracy among all the groups (68.9%), the percentage classified correctly is greater with the sexes separated than when the sexes are pooled. The testing accuracy for the male analysis demonstrate a notable decrease at 70.4%. One potential explanation is that the males in the testing sample may be more variable than the males in the training sample. Thus, the male-specific model is less proficient in generalizing to individuals that were not used to train the model, leading to increased misclassification. In particular, the coloured males in the testing model were observed to misclassify more frequently than was observed with the training model.

**Table 10:** Confusion matrix showing patterns of overlap and misclassification among the groups and sexes for the training model employing the MMS traits when separate sex-specific analyses are conducted

		Males				Females					
		Classifies into:			% Correct		Classifies into:				% Correct
**Group**:		BM	WM	CM		**Group**:		BF	WF	CF	
	BM	65	1	17	78.3		BF	61	3	19	73.5
	WM	3	77	3	92.8		WF	1	73	9	88.0
	CM	8	9	66	79.5		CF	19	7	57	68.9
Total:					83.5	Total:					76.7

## Discussion

Now more than ever, methods exploring population affinity need to be re-evaluated to ensure that valid methodology is employed, and that population variation is investigated and described in a scientifically meaningful way that offers valuable contributions to the community. As recommended by international standards of best practice, the estimation of population affinity should be based on peer-reviewed, published, and validated methods that make use of appropriate reference samples. The current study externally validates the MMS traits as a potential tool to estimate population affinity in South African anthropological analyses by providing population-specific data combined with robust quantitative analyses yielding high accuracies.

The variation observed among the three South African population groups has previously been discussed in terms of their population histories, which were significantly influenced by migration, colonization, and institutionalized racism [26, 28]. The current study revealed substantial group overlap in the crania of modern black, white and coloured South Africans. The MMS data demonstrate similar patterns of misclassification among the groups as documented in previous studies, where coloured South Africans misclassify nearly equal with both black and white South Africans [7, 8, 18]. In contrast, black and white South Africans rarely misclassified as one another. Coloured South Africans are typically reported to exhibit the lowest classification accuracy when compared to black and white South Africans, particularly in cranial analyses. This increased misclassification has been linked to their complex genetic composition [40], and the intermediacy in terms of cranial morphology relative to the other groups. Coloured South Africans have been shown to share similarities with white South Africans in cranial size but display greater similarities with black South Africans in terms of cranial shape [26, 28]. Despite the substantial overlap, various MMS traits demonstrated significant differences across all three groups, implying the potential for group differentiation when employed in multivariate analyses. The findings of the current study confirm the premise that the midface, and specifically the nasal region, plays a pivotal role in population affinity estimation. The midfacial variables not only demonstrated significant differences, with many showing marked differences among all three groups assessed, but also proved to be crucial within the classification models with the greatest values of variable importance. The MMS model outperformed measurement models from previously studies for the classification of the South African groups using standard craniometrics with discriminant analysis [7]. This is likely because much of the variation associated with the cranium is not quantified effectively when applying linear distances to measure a round object. The insights provided by the MMS traits regarding classification and relationships among population groups appear to be quite similar to those provided by craniometric data. Craniometric data has been demonstrated to be reliable proxies for neutral genetic information and population history, leading to greater confidence and acceptance of its use to estimate population affinity [41, 42]. Indeed, further research is needed to better understand the expression, ontogeny, and development of the MMS traits, as well as their relationship and covariation with craniometric data [43]. However, the results of this study challenge the notion that MMS traits should be excluded from population affinity estimation in forensic analyses [44]. Many authors have documented the superior results attainable through mixed models incorporating both metric and morphoscopic data [e.g., 45–47]. This approach warrants further investigation, not only to enhance the refinement of the MMS method but also to improve our comprehension of cranial variation.

Although the current study focused on large-scale population differences, the effects of sex on the classification of population affinity was also assessed. Although cranial sexual dimorphism of South Africans have been previously explored for the purpose of sex estimation [e.g., 48–51], few studies have compared sexual dimorphism among multiple different population groups simultaneously. Thus, there is a paucity of research that comprehensively assess the interaction of sex and population affinity on cranial morphology and its effects on the positive predictive performance of the cranium in correctly assigning sex and population affinity. In a morphoscopic study, Krüger et al. [52] identified significant differences between black and white South Africans using the Walker [53] traits, and thus supported the need for population-specific standards to estimate sex. L’Abbé and colleagues [7] simultaneously considered sex and population among South Africans when attempting to estimate population affinity with craniometrics and observed individuals more frequently misclassified as the incorrect sex rather than misclassifying as an incorrect population group. Concerning the MMS traits, Hefner [12] reported no significant sex differences, suggesting that the sexes be pooled for further analyses. However, sex has previously been shown to have a significant impact on inter-orbital breadth (IOB) in a South African population [18]. Similarly, the current study observed significant sex differences for several traits, including the inferior nasal margin (INA), inter-orbital breadth (IOB), malar tubercle (MT), nasal aperture width (NAW), posterior zygomatic tubercle (PZT), and supra-nasal suture (SPS). The current study also observed a tendency for the crania to misclassify according to sex, which was somewhat mitigated with the sex-specific analyses. Prior knowledge of sex has been shown to enhance classification accuracy in a South African sample by allowing classification models to focus solely on assessing differences related to population affinity, thereby reducing group overlap and facilitating more effective group separation [54]. Sexual dimorphism should be considered when exploring population variation, as the concepts of sexual dimorphism and population affinity are intricately linked.

This study supports previous research in stating the great potential of RFM as a classification method [45–47, 55]. As RFM is non-parametric, the method does not rely on statistical assumptions like normality, which are rarely met in real-world data. The method is capable of combining different types of data, and includes internal validation functionality which eliminates the need for additional independent samples to test the model validity. Finally, RFM is not prone to overfitting and the curse of dimensionality, which is a well-known issue encountered with discriminant analysis [56]. With discriminant analysis the inclusion of a greater number of measurements is typically recognized to allow more differences to be detected among groups. However, a decrease in classification accuracy will often be noted as more variables are added [57]. Essentially, redundant and highly correlated variables introduce statistical “noise”, which adversely affects the predictive performance of a model. The solution to this problem is to reduce the number of variables (typically done with stepwise variable selection) so that only the most discriminatory variables are retained [56, 57]. RFMs are capable of handling large numbers of variables, and it has been recommended that as many variables as possible be included and the model be allowed to run with them [14, 55]. Navega and colleagues [55] specifically caution against removing variables, even if they exhibit low measures of variable importance. Variable importance reflects the contribution of a specific trait or measurement to the overall ensemble of trees used in the model. However, each individual tree employs a random subset of variables at each split. Consequently, the overall contribution to the model may appear small, but the variable importance does not necessarily reflect how discriminative a variable can be for certain individual trees within the ensemble [55]. The current study demonstrated that the removal of even a single variable led to decreased accuracy. A notable strength of RFM is its efficiency in capturing interactions between variables as the model tests different combinations at each split, which makes it a highly effective classification tool with strong generalization capabilities [55].

A limitation of this study, and of MMS traits in general, is observer repeatability. Specifically, three traits, inferior nasal margin (INA), nasal overgrowth (NO), and nasal bone shape (NBS), demonstrated moderate repeatability, which is the lowest level of agreement recorded for the intra-observer analysis. Additionally, nasal bone contour (NBC) demonstrates slight agreement for the inter-observer comparison. This poses a potential issue, considering the high rankings of both INA and NBC in the classification model, and may impact predictive performance. Although the intra- and inter-observer agreement rates are consistent with those reported in other studies [12, 16–18], further efforts are needed to enhance trait repeatability before widespread use of the method in skeletal analyses in South Africa.

## Conclusion

The current study is the first to conduct a comprehensive analysis of MMS variation and predictive performance in a modern South African population. Numerous exploratory analyses were conducted to show that despite substantial heterogeneity and overlap, sufficient cranial differences exist among black, white and coloured South Africans to be able to estimate population affinity using the MMS traits. Ultimately, the classification models demonstrated that MMS traits outperform standard craniometric techniques currently employed for population affinity estimation. This confirms that the variation in the craniofacial complex results from both size and shape differences, an aspect more effectively quantified with MMS traits compared to linear cranial measurements, which predominantly assess size. The findings validate the use of MMS traits as a potential tool to estimate population affinity in South Africa. However, the low repeatability of some traits is of concern and requires further work to ensure more reliable results when conducting skeletal analyses.

**Declarations**.

## Data Availability

The dataset generated/analysed during the current study are available from the corresponding author on reasonable request.
